# Supplementary material for: Evaluation of renal markers in systemic autoimmune diseases
Source: PLoS One. 2023 Jun 23;18(6):e0278441. doi: 10.1371/journal.pone.0278441 (PMC10289317; doi:10.1371/journal.pone.0278441)
Supplement: S1 File — (DOCX) [file pone.0278441.s001.docx]

**Supplement Information I**

**Measurement of Renal Functional Panel Markers**

The Renal function panel included electrolytes (Sodium, Potassium, Chloride, Total Bicarbonate), minerals (Calcium, Phosphorus, Magnesium), protein (Albumin, Cystatin C), waste products (BUN, Creatinine), energy source (Glucose), and two calculated values (BUN/creatinine ratio, estimated glomerular filtration rate (eGFR)). These markers were quantitatively determined in Vibrant America, a CLIA-certified clinical laboratory. Most assays were quantitatively determined on Roche/Hitachi cobas c system if not mentioned specifically.

BUN in serum was measured via hydrolyzing urea by urease to form ammonium and carbonate. 2-oxoglutarate reacts with ammonium in the presence of glutamate dehydrogenase (GLDH) and the coenzyme NADH to produce L-glutamate. The rate of decrease in the NADH concentration was directly proportional to the urea concentration in the specimen and was measured photometrically.

Creatinine was measured by a kinetic colorimetric assay based on the Jaffé method. In alkaline solution, creatinine formed a yellow-orange complex with picrate. The rate of dye formation was proportional to the creatinine concentration in the specimen. To correct for non-specific reaction caused by serum/plasma pseudo-creatinine chromogens, including proteins and ketones, the results for serum or plasma were corrected by -26 µmol/L (-0.3 mg/dL).

Cystatin C was measured by latex enhanced immunoturbidimetric method on Beckman Counter AU series Analyzers. Cystatin C in the sample bind to the specific anti-Cystatin C antibody, which was coated on latex particles, and caused agglutination. The degree of the turbidity caused by agglutination can be measured optically and was proportional to the amount of Cystatin C in the sample.

Albumin was measured at a pH value of 4.1, in which it displayed a sufficiently cationic character to be able to bind with bromcresol green (BCG), an anionic dye, to form a blue-green complex. The color intensity of the blue-green color was directly proportional to the albumin concentration in the sample and was measured photometrically.

Electrolyte (sodium, potassium and chloride) were measured using Ion-Selective Electrode. The complete measurement system for a particular ion included the ISE, a reference electrode and electronic circuits to measure and process the EMF to give the test ion concentration. The sodium[^[i]^](https://inc-word-edit.officeapps.live.com/we/wordeditorframe.aspx?new=1&ui=en%2DGB&rs=en%2DUS&wopisrc=https%3A%2F%2Fvitasofttechnologies-my.sharepoint.com%2Fpersonal%2Fchithra_s_vitasoft-tech_com%2F_vti_bin%2Fwopi.ashx%2Ffiles%2F80a6bffe5b6f4aafb6b01e0b9d266cee&wdnewandopenct=1686724636110&wdprevioussession=018a3093-4f79-49c3-8104-5a4ea656c7f0&wdorigin=OFFICECOM-HWA.MAIN.NEW&wdenableroaming=1&mscc=1&wdodb=1&hid=C5ACBCA0-D032-2000-55ED-47CCF7486C62&jsapi=1&jsapiver=v1&newsession=1&corrid=35872b5c-3a42-499b-a208-a6b738ad0765&usid=35872b5c-3a42-499b-a208-a6b738ad0765&sftc=1&cac=1&mtf=1&sfp=1&wdredirectionreason=Unified_SingleFlush&rct=Normal&ctp=LeastProtected#_edn1) and potassium[^[ii]^](https://inc-word-edit.officeapps.live.com/we/wordeditorframe.aspx?new=1&ui=en%2DGB&rs=en%2DUS&wopisrc=https%3A%2F%2Fvitasofttechnologies-my.sharepoint.com%2Fpersonal%2Fchithra_s_vitasoft-tech_com%2F_vti_bin%2Fwopi.ashx%2Ffiles%2F80a6bffe5b6f4aafb6b01e0b9d266cee&wdnewandopenct=1686724636110&wdprevioussession=018a3093-4f79-49c3-8104-5a4ea656c7f0&wdorigin=OFFICECOM-HWA.MAIN.NEW&wdenableroaming=1&mscc=1&wdodb=1&hid=C5ACBCA0-D032-2000-55ED-47CCF7486C62&jsapi=1&jsapiver=v1&newsession=1&corrid=35872b5c-3a42-499b-a208-a6b738ad0765&usid=35872b5c-3a42-499b-a208-a6b738ad0765&sftc=1&cac=1&mtf=1&sfp=1&wdredirectionreason=Unified_SingleFlush&rct=Normal&ctp=LeastProtected#_edn2) electrodes were based on neutral carriers and the chloride[^[iii]^](https://inc-word-edit.officeapps.live.com/we/wordeditorframe.aspx?new=1&ui=en%2DGB&rs=en%2DUS&wopisrc=https%3A%2F%2Fvitasofttechnologies-my.sharepoint.com%2Fpersonal%2Fchithra_s_vitasoft-tech_com%2F_vti_bin%2Fwopi.ashx%2Ffiles%2F80a6bffe5b6f4aafb6b01e0b9d266cee&wdnewandopenct=1686724636110&wdprevioussession=018a3093-4f79-49c3-8104-5a4ea656c7f0&wdorigin=OFFICECOM-HWA.MAIN.NEW&wdenableroaming=1&mscc=1&wdodb=1&hid=C5ACBCA0-D032-2000-55ED-47CCF7486C62&jsapi=1&jsapiver=v1&newsession=1&corrid=35872b5c-3a42-499b-a208-a6b738ad0765&usid=35872b5c-3a42-499b-a208-a6b738ad0765&sftc=1&cac=1&mtf=1&sfp=1&wdredirectionreason=Unified_SingleFlush&rct=Normal&ctp=LeastProtected#_edn3) electrode was based on an ion exchanger.

Total bicarbonate in sample reacted with phosphoenolpyruvate (PEP) in the presence of PEPC to produce oxaloacetate and phosphate: This reaction was coupled with one involving the transfer of a hydrogen ion from NADH analog to oxaloacetate using MDH. The resultant consumption of NADH analog caused a decrease in absorbance, which was proportional to the concentration of bicarbonate in the sample being assayed.

Calcium in serum was measured by reacting with 5-nitro-5’-methyl-BAPTA (NM-BAPTA) under alkaline conditions to form a complex. This complex reacted with EDTA. The change in absorbance was directly proportional to the calcium concentration and was measured photometrically.

Phosphate was measured by Molybdate UV. Inorganic phosphate formed an ammonium phosphomolybdate complex having the formula (NH_4_)_3_[PO_4_(MoO_3_)_12_] with ammonium molybdate in the presence of sulfuric acid. The concentration of phosphomolybdate formed was directly proportional to the inorganic phosphate concentration and was measured photometrically.

Magnesium was measured in alkaline solution, forming a purple complex with xylidyl blue, diazonium salt. The magnesium concentration was measured photometrically via the decrease in the xylidyl blue absorbance.

Glucose was measured by UV test. Hexokinase catalyzed the phosphorylation of glucose to glucose-6-phosphate by ATP. Glucose-6-phosphate dehydrogenase oxidized glucose-6-phosphate in the presence of NADP to gluconate-6-phosphate. The rate of NADPH formation during the reaction was directly proportional to the glucose concentration and was measured photometrically.

References

[^[i]^](https://inc-word-edit.officeapps.live.com/we/wordeditorframe.aspx?new=1&ui=en%2DGB&rs=en%2DUS&wopisrc=https%3A%2F%2Fvitasofttechnologies-my.sharepoint.com%2Fpersonal%2Fchithra_s_vitasoft-tech_com%2F_vti_bin%2Fwopi.ashx%2Ffiles%2F80a6bffe5b6f4aafb6b01e0b9d266cee&wdnewandopenct=1686724636110&wdprevioussession=018a3093-4f79-49c3-8104-5a4ea656c7f0&wdorigin=OFFICECOM-HWA.MAIN.NEW&wdenableroaming=1&mscc=1&wdodb=1&hid=C5ACBCA0-D032-2000-55ED-47CCF7486C62&jsapi=1&jsapiver=v1&newsession=1&corrid=35872b5c-3a42-499b-a208-a6b738ad0765&usid=35872b5c-3a42-499b-a208-a6b738ad0765&sftc=1&cac=1&mtf=1&sfp=1&wdredirectionreason=Unified_SingleFlush&rct=Normal&ctp=LeastProtected#_ednref1) Shono T, Okahara M, Ikeda I, et al. Sodium-selective PVC Membrane Electrodes Based on Bis(12-crown-4)s. J Electroanal Chem 1982;132:99-105.

[^[ii]^](https://inc-word-edit.officeapps.live.com/we/wordeditorframe.aspx?new=1&ui=en%2DGB&rs=en%2DUS&wopisrc=https%3A%2F%2Fvitasofttechnologies-my.sharepoint.com%2Fpersonal%2Fchithra_s_vitasoft-tech_com%2F_vti_bin%2Fwopi.ashx%2Ffiles%2F80a6bffe5b6f4aafb6b01e0b9d266cee&wdnewandopenct=1686724636110&wdprevioussession=018a3093-4f79-49c3-8104-5a4ea656c7f0&wdorigin=OFFICECOM-HWA.MAIN.NEW&wdenableroaming=1&mscc=1&wdodb=1&hid=C5ACBCA0-D032-2000-55ED-47CCF7486C62&jsapi=1&jsapiver=v1&newsession=1&corrid=35872b5c-3a42-499b-a208-a6b738ad0765&usid=35872b5c-3a42-499b-a208-a6b738ad0765&sftc=1&cac=1&mtf=1&sfp=1&wdredirectionreason=Unified_SingleFlush&rct=Normal&ctp=LeastProtected#_ednref2) Shibata Y, Maruizume T, Miyage H. Journal of the Chemical Society of Japan. Chemistry and Industrial Chemistry. 1992;9:961-967.

[^[iii]^](https://inc-word-edit.officeapps.live.com/we/wordeditorframe.aspx?new=1&ui=en%2DGB&rs=en%2DUS&wopisrc=https%3A%2F%2Fvitasofttechnologies-my.sharepoint.com%2Fpersonal%2Fchithra_s_vitasoft-tech_com%2F_vti_bin%2Fwopi.ashx%2Ffiles%2F80a6bffe5b6f4aafb6b01e0b9d266cee&wdnewandopenct=1686724636110&wdprevioussession=018a3093-4f79-49c3-8104-5a4ea656c7f0&wdorigin=OFFICECOM-HWA.MAIN.NEW&wdenableroaming=1&mscc=1&wdodb=1&hid=C5ACBCA0-D032-2000-55ED-47CCF7486C62&jsapi=1&jsapiver=v1&newsession=1&corrid=35872b5c-3a42-499b-a208-a6b738ad0765&usid=35872b5c-3a42-499b-a208-a6b738ad0765&sftc=1&cac=1&mtf=1&sfp=1&wdredirectionreason=Unified_SingleFlush&rct=Normal&ctp=LeastProtected#_ednref3) Hartman K, Luterotti S, Osswald HF, et al. Chloride-selective liquid-membrane electrodes based on lipophilic methyl-tri-N-alkyl-ammonium compounds and their applicability to blood serum measurements. Microchimica Acta 1978;70(3-4):235-246.
