# Supplementary material for: Evaluation of renal markers in systemic autoimmune diseases
Source: PLoS One. 2023 Jun 23;18(6):e0278441. doi: 10.1371/journal.pone.0278441 (PMC10289317; doi:10.1371/journal.pone.0278441)
Supplement: S2 File — (DOCX) [file pone.0278441.s002.docx]

**Supplement Information II**

**Reference Range for Normal Levels of Renal Markers**

| **Renal Marker** | **Age** | **Normal Reference Range** |
| --- | --- | --- |
| Chloride | All ages | 98~107 mmol/L |
| Carbon Dioxide | Adult | 18~29 mmol/L |
| Carbon Dioxide | Children | 23~30 mmol/L |
| Potassium | Adult | 3.5~5.1 mmol/L |
| Potassium | Children | 3.5~5.5 mmol/L |
| Sodium | All ages | 136~145 mmol/L |
| Phosphate, Inorganic | Adult | 2.5~4.5 mg/dL |
| Phosphate, Inorganic | Children | 2.5~4.5 mg/dL |
| Glucose | Adult | 70~100 mg/dL |
| Glucose | Teenager | 70~99 mg/dL |
| Glucose | Children | 65~110 mg/dL |
| Glucose | Baby | 60~115 mg/dL |
| Creatinine (female) | All ages | 0.5~0.9 mg/dL |
| Creatinine (male) | All ages | 0.7~1.2 mg/dL |
| BUN | >60 years old | 8~23 mg/dL |
| BUN | Adult | 6~20 mg/dL |
| BUN | Children | 5~23 mg/dL |
| Cystatin C | All ages | 0.62~1.16 mg/L |
| Magnesium | All ages | 1.6~2.6 mg/dL |
| Calcium | All ages | 8.9~10.6 mg/dL |
| Albumin | All ages | 3.5~5.2 g/dL |
| eGFR | Adult | 0~0 mL/min/1.73m^2^ |
| eGFR | Children | 60~1.798e308 mL/min/1.73m^2^ |
| BUN/Creatinine Ratio | All ages | 10~20 |
