# Supplementary material for: Evaluation of renal markers in systemic autoimmune diseases
Source: PLoS One. 2023 Jun 23;18(6):e0278441. doi: 10.1371/journal.pone.0278441 (PMC10289317; doi:10.1371/journal.pone.0278441)
Supplement: Attachment — Submitted filename: Manuscript Review.docx [file pone.0278441.s003.docx]

**Manuscript Review**

**Manuscript ID:** PONE-D-22-31509

**Title:** Evaluation of Renal Markers in Systemic Autoimmune Diseases

**Journal**: PloS One

**Summary and general comments:**

In the manuscript “**Evaluation of Renal Markers in Systemic Autoimmune Diseases**” the authors took a correlation approach to investigate the association between several renal markers and kidney disease in patients with systemic autoimmune diseases. To achieve such aim, the authors compared retrospectively 13 renal markers together with autoantibodies in 3 cohorts of patients: 1) 126 with systemic lupus erythematosus (SLE), 2) 695 with connective tissue diseases (CTDs) and 3) 3304 with rheumatoid arthritis (RA).

The results showed that:

1. Renal markers were elevated in the 3 cohorts of patients comparatively to controls.
2. Some autoantibodies such ANA, anti-Histone and anti-CCP were associated with kidney damage and lower eGFRs.
3. All the renal markers except for electrolytes were good in assessing renal involvement in patients with autoimmune diseases.

Overall, the paper is well organized and I enjoyed reading it but there are some rectifications and explanations that need to be addressed.

**Specific comments:**

**#1** In the text, the authors mention 4 tables that do not exist in the manuscript version submitted for review. Please provide the manuscript with the cited tables.

**#2** The last paragraph of the introduction is a bit misleading. For instance, the authors mention 13841 SLE patients when in reality it is 126 patients and 13715 controls. And then who were these control subjects, and why did each of the 3 cohorts have its own control group?

**#3** In the statistical analysis section, the authors did not specify the type of statistical tests used.

**#4** In the results section, the authors did not detail the quantitative measurements of the different markers measured in the patients. It would be more appropriate to compare the means or to perform non-parametric tests followed by ROC analysis to evaluate the sensitivity and specificity of the markers in detecting renal damage. Eventually, the authors could perform a logistic regression to estimate the incremental risk for each one-unit increase in each marker and build a predictive model for kidney damage.

**#5** In SLE patients, the association of anti-DNA antibodies with renal damage has been known for a long time and numerous human and mouse studies have confirmed the pathogenic role of these autoantibodies. The authors should discuss the discrepancy of their results with the literature.
